# Applications of Acupuncture Therapy in Modulating the Plasticity of Neurodegenerative Disease and Depression: Do MicroRNA and Neurotrophin BDNF Shed Light on the Underlying Mechanism?

**DOI:** 10.1155/2020/8850653

**Published:** 2020-09-22

**Authors:** Xia Li, Jun Zhao, Zhigang Li, Li Zhang, Zejun Huo

**Affiliations:** ^1^School of Acupuncture-Moxibustion and Tuina, Beijing University of Chinese Medicine, Beijing 100029, China; ^2^Department of Chinese Medicine, Peking University 3rd Hospital, Beijing 100191, China

## Abstract

As the global population ages, the incidence of neurodegenerative diseases has risen. Furthermore, it has been suggested that depression, especially in elderly people, may also be an indication of latent neurodegeneration. Stroke, Alzheimer's disease (AD), and Parkinson's disease (PD) are usually accompanied by depression. The urgent challenge is further enforced by psychiatric comorbid conditions, particularly the feeling of despair in these patients. Fortunately, as our understanding of the neurobiological substrates of maladies affecting the central nervous system (CNS) has increased, more therapeutic options and novel potential biological mechanisms have been presented: (1) Neurodegenerative diseases share some similarities in their pathological characteristics, including changes in neuron structure or function and neuronal plasticity. (2) MicroRNAs (miRNAs) are small noncoding RNAs that contribute to the pathogenesis of diverse neurological disease. (3) One ubiquitous neurotrophin, brain-derived neurotrophic factor (BDNF), is crucial for the development of the nervous system. Accumulating data have indicated that miRNAs not only are related to BDNF regulation but also can directly bind with the 3′-UTR of BDNF to regulate BDNF and participate in neuroplasticity. In this short review, we present evidence of shared biological substrates among stroke, AD, PD, and depression and summarize the possible influencing mechanisms of acupuncture on the neuroplasticity of these diseases. We discuss neuroplasticity underscored by the roles of miRNAs and BDNF, which might further reveal the potential biological mechanism of neurodegenerative diseases and depression by acupuncture.

## 1. Introduction

Neurodegenerative diseases, including stroke, Alzheimer's disease (AD), and Parkinson's disease (PD), are chronic progressive diseases caused by the apoptosis, loss, and degeneration of neurons in the central nervous system (CNS) and alter neuroplasticity [1]. Moreover, major depressive disorder has strong relationships with neurodegenerative diseases and the natural processes of ageing: they have not only overlapping clinical features, such as mood disorder [2], but also neuroplasticity mediated by neurotrophic factors that also orchestrate adaptive defensive behaviours [3]. Hence, the links among brain plasticity, neurodegenerative diseases, and depression are of interest to researchers [4].

Neuroplasticity is the ability of the brain's neural network system to adapt to changes in internal and external environments and to alter the structure and function of neurons accordingly. In other words, neuroplasticity can be defined as “the ability of the nervous system to respond to intrinsic or extrinsic stimuli by reorganizing its structure, connections, and function” [5]. Neuroplasticity includes structural plasticity and functional plasticity of the nervous system, which makes up the physiological basis for repair when the nervous system is damaged [6]. Brain structural plasticity is an extraordinary tool that allows the mature brain to adapt to environmental changes and repair itself after lesions or disease and slow ageing. Its function involves behavioural performance, learning and memory, mental activity, and other neurobiological processes.

Brain-derived neurotrophic factor (BDNF) stands out due to its high level of expression in the brain and its potent effects on synapses [7]. BDNF regulates the structural plasticity of nerves not only by promoting the growth, reconstruction, and synaptic formation of axons and dendrites but also by changing synaptic transmission and affecting the functional plasticity of nerves through presynaptic and postsynaptic mechanisms. BDNF also regulates activity-dependent forms of synaptic plasticity, such as long-term potentiation (LTP), which is thought to underlie learning and memory [8]. Simultaneously, converging evidence strongly suggests that deficits in BDNF signalling or decreased BDNF leads to the pathogenesis of several major diseases and disorders, such as AD and PD [7, 9]. BDNF has emerged as a key facilitator of neuroplasticity involved in motor learning and rehabilitation after stroke [10]. In addition, BDNF has been shown to be critically involved in the regulation of synaptic plasticity and the pathophysiology of mood disorders [11, 12]. Remarkably, according to the published literature, AD, stroke, and PD are usually accompanied by depression, and these neurodegenerative diseases and depression are related to BDNF. If a common way to regulate BDNF was determined, it could be used to regulate neuroplasticity to delay the progression of diseases.

Fortunately, emerging studies have shown that microRNAs (miRNAs) not only contribute to the pathogenesis of neurological disease but also play important roles in neurogenesis, neurodevelopment, and neural plasticity [13, 14]. miRNAs can posttranscriptionally degrade mRNA or inhibit the translation of mRNA by binding the 3′-UTR section of mRNAs, further influencing the expression of target genes [15]. In a limited number of studies published thus far, we searched electronic bibliographic databases and found that some miRNAs not only are related to BDNF regulation but also can directly bind with the 3′-UTR of BDNF to regulate BDNF and participate in neuroplasticity [16]. Hence, miRNA/BDNF regulatory networks may be closely related to neural plasticity. Numerous previous studies have shown that acupuncture has positive clinical effects on stroke, AD, PD, and depression. We reviewed recent publications related to acupuncture on related miRNAs and BDNF in neurodegenerative diseases and depression. The aim of this study was to explore the biological mechanisms underlying the comorbidity of these diseases and the effect of acupuncture on regulating neural plasticity.

## 2. Main Text

### 2.1. Stroke

#### 2.1.1. BDNF Plays Important Roles in Stroke

A previous study identified that several therapeutic interventions, such as exercise and rehabilitation, enhance functional recovery after stroke. The beneficial effects of these therapies include improved learning and memory, improved motor function, and increased expression of proteins involved in brain plasticity, such as BDNF [17]. A clinical study showed an increased number of BDNF-producing Treg cells after stroke, suggesting the possibility that Treg cells may be able to supply BDNF to the site of injury to confer neuroprotection after stroke [18]. Similarly, in middle cerebral artery occlusion (MCAO) model rats, strategies that widely increase BDNF within the nervous system were found to enhance neuroplasticity processes involved in motor relearning during stroke rehabilitation, whereas attenuating BDNF levels in the brain completely negated the recovery of skilled motor movements [19]. Hence, capitalizing on the beneficial effects of BDNF in the CNS may be effective for facilitating recovery after stroke.

#### 2.1.2. miRNAs Play Important Roles in Stroke

miRNAs are increasingly believed to play important roles in neuroprotection and synaptic plasticity during and after ischaemia. For instance, miR-124 is highly specific to neurons in cerebral ischaemic injury and may play a dual role in regulating apoptosis and exerting detrimental effects on synaptic plasticity and axonal growth [20]. Upregulation of miR-191a-5p exacerbated neuronal injury in ischaemic stroke. Conversely, downregulation of miR-191a-5p expression in the cortex partly reversed this injury [21]. Similarly, miR-195 downregulated Kruppel-like factor 5 (KLF5) and blocked the JNK signalling pathway, ultimately inhibiting neuronal apoptosis in rats with ischaemic stroke [22]. Additionally, miR-133b can regulate gene expression, promote neurite remodelling, and improve functional recovery in rats subjected to MCAO [23]. Intracerebroventricular injection of miR-494 agomir reduced neuronal apoptosis and infarct volume during the acute stage of MCAO and promoted axonal plasticity and long-term outcomes during the recovery stage [24]. Similarly, miR-181a can regulate synaptic function in stroke recovery, and the dendrites of miR-181a-overexpressing neurons have fewer and smaller spines [25]. Consistent with these general observations, miR-134 was enriched in the neuronal dendrites of a rat model of stroke hippocampal CA1 and negatively controlled the size of dendritic spines; thus, regulating synaptic-dendritic plasticity may ameliorate cognitive impairment in rats with MCAO-induced cognitive deficits [26]. According to the above results, miRNAs could potentially predict stroke outcomes as novel biomarkers.

#### 2.1.3. miRNAs May Regulate Stroke via Influencing BDNF

The miRNA-related BDNF signalling pathway plays a significant role in the pathogenesis of stroke and seems to be a promising therapeutic target, as summarized in Table 1. In acute ischaemic stroke patients, miR-124 was targeted by the 3′-UTR of BDNF mRNA [27]. Additionally, a dual-luciferase reporter assay identified BDNF as the direct target of miR-210, which is a crucial ischaemic stroke-associated miRNA and a potential target for stroke therapy [28]. Interestingly, the SNP rs7124442 in the 3′-UTR of BDNF might also act as a protective factor in patients with ischaemic stroke by affecting the regulatory role of miR-922 in BDNF expression [29]. Similarly, in MCAO brain tissues, bioinformatic analysis showed that miR-10b-5p could bind directly to the 3′-UTR sites of BDNF and negatively regulate its expression [30]. In a recent study, miR-155 targeted BDNF, and downregulation of miR-155-targeted BDNF transcripts protected against ischaemic brain injury [31]. Another study reported a similar conclusion: miR-155, miR-1, miR-10b, and miR-191 directly repressed BDNF by binding to their predicted sites in the 3′-UTR of BDNF [32]. Although we found that miR-9 regulates axon extension and branching by targeting Map 1b (not BDNF) in mouse cortical neurons, the associations are intriguing (short stimulation with BDNF decreases miR-9 expression, whereas prolonged stimulation with a high concentration of BDNF increases miR-9 expression in the axon) [33]. The potential regulatory signalling pathway between miRNA and BDNF acts in a biphasic manner and is worthy of being analysed and studied further.

#### 2.1.4. Acupuncture Plays a Therapeutic Role in Stroke by Regulating the Expression of BDNF and miRNAs

Previous studies have shown that the number of BDNF-positive neurons or neurons with localized BDNF expression was downregulated in the peri-infarct cortex, the striatum, the subventricular zone, and the hippocampus of ischaemia and reperfusion- (I/R-) injured rats [34, 35]. However, Jiang reported that electroacupuncture (EA) can increase the synthesis and release of BDNF after ischaemia [36]. As summarized in Table 1, Min et al. reported that EA at GV20 increased the expression of BDNF associated with motor recovery [37]. Zhou further explored a possible compensatory part of the functional mechanism of EA that involves regulation of the contralateral cerebral cortex. It was revealed that EA can improve the symptoms of neurological deficits and motor function recovery in rats [38]. In addition, Teng showed the mechanism of acupuncture at the GV20-GB7 scalp cave on intracerebral haemorrhage. According to their results, acupuncture can play a role in protecting the brain [39]. Similarly, nape cluster acupuncture exerts protective and reparative effects on the brain tissue in rats with postischaemic stroke sequelae [40]. EA was administered at acupoints LI11 and ST36 to promote the repair of ischaemic injured neurons and reduce their apoptosis [41]. The above studies all showed that after administration of acupuncture, neurological deficits and cerebral infarcts were also improved, and the mechanism of action of EA may involve effective upregulation of rat brain tissue BDNF protein expression.

Many studies have reported how acupuncture can regulate the expression of miRNAs in stroke. Liu published a study in which a bioinformatic analysis of 48 miRNAs in the ischaemic hippocampus CA1 was used to test the underlying mechanism of EA in ischaemic stroke. According to the results, miR-132, miR-134, miR-125b, miR-181a, etc. were downregulated, which was related to learning memory. In addition, miR-219a, which is closely related to synaptic plasticity, was also downregulated by EA treatment at the GV20 and GV24 acupoints [42]. However, upregulation of miR-191a-5p exacerbated neuronal injury and partly reversed the neuroprotective effect of EA treatment after I/R injury [21]. Zhao et al. aimed to identify whether upregulation of miR-132 by EA improved the damaged nerves after stroke. After administration of EA, upregulated miR-132 suppressed SOX2 in primary neurons after oxygen-glucose deprivation, which promoted neurite outgrowth [43]. Liu et al. found that the density of dendritic spines and the number of synapses in hippocampal CA1 pyramidal cells were obviously reduced in stroke model rats. In this study, EA decreased the expression of miR-134, thereby negatively regulating LIMK1 to enhance synaptic-dendritic plasticity [26]. Intriguingly, Liu et al. also investigated the neuroprotective mechanism of miR-9-mediated activation of the nuclear factor-*κ*B signalling pathway by EA at acupoints LI11 and ST36. Compared with pre-EA treatment conditions, the expression of miR-9 in the peri-infarct cortex was increased; conversely, miR-9 inhibitors suppressed the cerebral protective efficacy of EA treatment [44]. Zheng et al. evaluated changes in the cerebral cortical miRNA profile, cerebral blood flow, and neurological function induced by EA in a rat model of stroke. In that study, miR-494 was downregulated, and miR-206 was upregulated in the penumbra. Simultaneously, EA increased cerebral blood flow and alleviated neurological impairment in rats [45]. Similarly, Deng et al. treated acupoint GV20 after ischaemic stroke. EA increased miR-181b levels in the penumbras and improved neurobehavioural function [46].

In summary, the above studies indicated that acupuncture may play an important role in the neural plasticity of stroke by regulating the expression levels of miRNAs and BDNF. These studies also suggest that epigenetic regulation is critical for synaptic plasticity and warrants specific investigations in the setting of stroke recovery.

### 2.2. PD

#### 2.2.1. BDNF Plays Important Roles in PD

PD is a disabling neurodegenerative disease that may be associated with nonmotor symptoms, such as cognitive deficits, and is often accompanied by altered BDNF production [47]. Neurons expressing particularly low levels of BDNF may be at greatest risk of injury in PD and possibly a trigger for degeneration itself [48]. BDNF was positively correlated with a longer time span of disease, the severity of PD symptoms, and more advanced stages of disease [49]. These findings suggest that BDNF may be implicated in the pathogenic mechanisms of PD. Excitingly, in recent years, clinical studies have demonstrated that treatment with antiparkinsonian drugs may increase BDNF levels [50]. Similarly, exercise therapy can trigger several plasticity-related events in the human PD brain, including corticomotor excitation and changes in BDNF levels [51]. In general, BDNF may be a potential biomarker for evaluating cognitive changes in PD and other neurological syndromes associated with cognitive decline [47].

#### 2.2.2. miRNAs Play Important Roles in PD

Over the past decade, many studies have reported dysregulation of miRNA expression in PD [52]. Specific miRNAs of interest that have been implicated in PD pathogenesis include miR-29, miR-26, miR-485, miR-30, and let-7 [53]. For instance, miR-29 has been shown to regulate various processes that are important in PD development, such as apoptosis, neuronal survival, and epigenetic modulation [54]. Similarly, miR-26 can modulate processes such as DNA repair and LTP maintenance [55]. One study further identified a developmentally and activity-regulated miR-485 that controls dendritic spine number and synapse formation in an activity-dependent homeostatic manner [56]. Furthermore, miR-132 widely participates in axon growth, neural migration, and plasticity. However, dysregulation of miR-132 results in the occurrence and exacerbation of neural developmental degenerative diseases, such as AD and PD. Regulating miR-132 expression relieves symptoms, alleviates severity, and finally affects a cure [57]. Hence, it is important to identify and validate these miRNAs in the ageing PD brain.

#### 2.2.3. miRNAs May Regulate PD via Influencing BDNF

The analysis of miRNA expression in biopsy specimens from the PD brains combined with target mRNA identification might provide new therapeutic options. As summarized in Table 2, previous studies have shown that miR-494-3p plays a role in promoting the development of PD [58], and the online starBase database predicted the existence of complementary sequences between miR-494-3p and BDNF, indicating that BDNF might be a target of miR-494-3p. According to their results, abnormally expressed miR-494-3p and BDNF might be associated with the development of PD [59]. On the other hand, it has been reported that miR-30a-5p is a potential biomarker for PD [60], targeting and suppressing BDNF expression in the prefrontal cortex [61], and a lower BDNF level is associated with greater cognitive impairments in PD patients [9]. In addition, a recent study reported that miR-7 was upregulated in the brain tissue of rats with atrazine-induced PD. The study also identified that miR-7 regulated the expression of BDNF through an autoregulatory mechanism [62]. Similarly, it has been reported that miR-210-3p targets BDNF mRNA. Therefore, according to the study conclusion, interfering with miRNA expression could be a strategy for BDNF regulation in PD pathogenesis [63].

#### 2.2.4. Acupuncture Plays a Therapeutic Role in PD by Regulating the Expression of BDNF and miRNAs

Accumulating clinical evidence has shown that using EA as a complementary therapy ameliorates motor and nonmotor symptoms of PD and improves the plasticity of synaptic activity [64]. Acupuncture can cause changes in the neuroplasticity of PD, manifested by increasing BDNF expression levels and promoting nerve regeneration [65], as summarized in Table 2. On the one hand, BDNF may change the mechanism of synaptic plasticity, which is critical for cognition and memory. EA responsiveness to PD was studied by Huang et al., whereby EA was administered to acupoints LR3 and GV16 in a PD rat model. After EA treatment, learning and memory abilities were significantly improved, and BDNF was increased compared with the model group [66]. On the other hand, PD is characterized by dopaminergic neuron loss in the substantia nigra. EA therapy may attenuate this loss by promoting the expression of endogenous BDNF [67]. For instance, in a rat model of PD, EA treatment ameliorated motor impairments and dopaminergic neuron loss, and these changes were accompanied by significantly upregulated BDNF expression in both the substantia nigra and the striatum [68]. Furthermore, acupuncture, especially combined therapy with medoba, at the control area of dancing tremors in PD mice improved the absence of dopaminergic neurons in the substantia nigra by enhancing the expression of BDNF in the brain [69]. In addition, 6-hydroxydopamine (6-OHDA) lesion rat models of PD were used by Zhang, who reported that EA induced an increase in BDNF mRNA expression in PD model rats [70]. Moreover, Wang tested the effects of different amounts of electricity on the positive cell count of black striatum BDNF in PD model rats which were compared, and the related mechanism was discussed. According to their results, one of the therapeutic mechanisms of music and pulse EA in PD model rats was achieved by regulating the number of black striatum BDNF-positive cells [71]. Interestingly, Sun et al. and Liang et al. compared the effects of different frequencies of chronic EA stimulation in a rat model of PD. The results indicated that 4 weeks of EA treatment at 100 Hz reversed the 6-OHDA-induced abnormal expression of BDNF on the lesioned side in the ventral midbrain and the hippocampus [72]. Similarly, compared with pre-EA treatment conditions, the levels of BDNF mRNA in the SN and the ventral tegmental area of the lesioned side were significantly increased in the 100 Hz EA group but unchanged in the 0 and 2 Hz groups. The authors also suggested that activation of endogenous BDNF by long-term high-frequency EA may be involved in the regeneration of injured dopaminergic neurons, which may underlie the effectiveness of EA in the treatment of PD [73].

Previous studies have shown that miR-124 is closely related to PD [74], and the overexpression of miR-124 diminished the production of CDK5 by inhibiting the calpain1/p25/CDK5 pathway. Furthermore, CDK5 silencing could give rise to upregulated BDNF and relieve synaptic failure in PD [20]. Liu further studied whether acupuncture could regulate the expression of miR-124 in the striatum of transgenic mice with PD. Acupuncture was performed on acupoints GB34 and LR3. Compared with preacupuncture treatment conditions, the expression of miR-124 and BDNF protein was upregulated. The author also suggested that the miR-124/BDNF signalling pathway may be involved in the pathogenesis of PD [75].

To summarize, acupuncture treatment appears to be a promising approach for the management of PD. Acupuncture regulated miRNA levels and promoted BDNF expression, which seem to play important roles in the development of PD.

### 2.3. AD

#### 2.3.1. BDNF Plays Important Roles in AD

AD is a progressive neurodegenerative disorder resulting in memory loss and eventually dementia [76]. BDNF is required for learning and memory, and this crucial protein is significantly reduced in the brains of AD patients, leading to reduced plasticity and neuronal death [77]. Accumulating data have also indicated that there is a general reduction in BDNF mRNA and protein in AD animal models [76]. These findings have contributed to the development of BDNF treatment regimens for AD.

#### 2.3.2. miRNAs Play Important Roles in AD

There is now considerable evidence that the dysregulation of miRNAs correlates with the progression and severity of AD [78]. The differential expression of miRNAs has been reported in many brain regions [79]. miRNAs can also regulate synaptic transmission and plasticity in the hippocampus and neocortex and regulate memory formation [80]. It has been reported that miR-132 exerts neuroprotective function as it has been shown to regulate both neuron morphogenesis and plasticity, and it is the most significantly reduced miRNA in the brains of AD patients. Research has further confirmed that genetic deletion of miR-132 in mice promotes A*β* deposition, leading to impaired memory and enhanced Tau pathology [81]. However, the upregulation of miR-142-5p and miR-134-5p expression contributes to the pathogenesis of AD by triggering synaptic dysfunction associated with A*β*-mediated pathophysiology [79, 82]. In learning memory aspects, miR-124 and miR-181a, which are two miRNAs that are upregulated in the hippocampus, are directly associated with deficits in synaptic plasticity [83, 84]. Similarly, overexpression of miR-338-5p and miR-181 functionally prevented impairments in synaptic plasticity, learning ability, and memory retention in an animal model of AD [85, 86]. Furthermore, overexpression of miR-153 has provided new insight into the molecular mechanism of presynaptic plasticity impairment at the miRNA level and suggests that chronic brain hypoperfusion obstructs presynaptic vesicle fusion with the presynaptic membrane via miR-153-mediated downregulation of multiple synaptic vesicle-related proteins [87]. Previously, miR-34a and miR-34c were confirmed to be involved in synaptic deficits in AD pathological development, to influence synaptic plasticity and to play key roles in AD pathogenesis [88, 89]. Evidence from a recent study indicated that the miR-34a gene and miR-34a-mediated concurrent repression of its target genes in neural networks may result in dysfunction of synaptic plasticity, energy metabolism, and resting state network activity [90]. In addition, the dysregulation of certain miRNAs is also strongly correlated with the presence of AD-type neuropathological changes. There are notable miRNAs that are regulated in AD. For example, in postmortem AD brains, three miRNAs were upregulated—miR-30a-5p, miR-206, and miR-92b-3p [61, 91]—and four miRNAs were downregulated—miR-132/212 cluster, miR-9, miR-129, and miR-136 [78, 92]. In summary, our results provide insights into polygenetic AD mechanisms and reveal that miRNAs may be involved in neural plasticity as potential therapeutic targets for AD.

#### 2.3.3. miRNAs May Regulate AD via Influencing BDNF

The miRNA-related BDNF signalling pathway seems to be both profitable and promising for AD treatment, as summarized in Table 3. Two previous studies confirmed that BDNF exerts its beneficial effects on CNS neurons via upregulation of miR-132 [93, 94]. A later study pointed out that both AD patients and AD models have high levels of miR-206 in the brain, which contributes to memory impairments by suppressing the expression of BDNF [95, 96]. Similarly, recent evidence suggests that the miR-134-5p-mediated posttranscriptional regulation of CREB-1 and BDNF is an important molecular mechanism underlying plasticity deficits in AD [79]. Luciferase assays confirmed that miR-30a-5p, miR-195, and miR-613 can target specific sequences surrounding the proximal polyadenylation site within the BDNF 3′-untranslated region [61, 97]. Neuronal overexpression of miR-30a-5p resulted in downregulation of BDNF protein [61]. Another dual-luciferase reporter gene assay demonstrated that miR-10a targeted BDNF, and the authors indicated that miR-10a restrains synapse remodelling and neuronal cell proliferation while promoting apoptosis in AD rats by inhibiting the BDNF-TrkB signalling pathway [98]. Furthermore, miR-322 is significantly increased with the decrease in BDNF in the AD mouse brain, and a luciferase reporter assay identified that miR-322 can directly conjugate to the 3′-UTR of BDNF [16]. As such, there is a novel miRNA-dependent mechanism of BDNF degradation in AD pathogenesis, which may drive miRNA- or BDNF-based therapeutic strategies against AD.

#### 2.3.4. Acupuncture Plays a Therapeutic Role in AD by Regulating the Expression of BDNF

Previous studies have shown that electrotherapy can repair the synaptic form and inhibit synaptic degeneration of hippocampal neurons in AD rats [99]. More importantly, the efficacy of EA was demonstrated by regulating the expression of BDNF, as summarized in Table 3. Both studies showed that EA can upregulate the expression of hippocampal BDNF, maintain hippocampal LTP to a certain extent [100], and enhance neurogenesis to improve learning and memory in AD rats [101]. Similarly, Li et al. also showed that repeated EA stimulation may improve cognitive function, upregulate the expression of BDNF, and promote neurogenesis in AD [102]. Moreover, Lin et al. showed that EA at acupoint GV20 can significantly increase the expression levels of mature BDNF and a precursor protein, proBDNF, in APP/PS1 mice. EA may serve as a promising treatment strategy for AD, which may exert neuroprotective effects by adjusting the expression and processing of BDNF [103].

Intriguingly, Keifer et al. published a study aimed at exploring the interrelationship of the miRNA-BDNF signalling loop in the AD brain. According to their results, the reduction in BDNF that occurs in the AD brain is the result of two independent mechanisms: (1) a failure in the proteolytic conversion of BDNF precursor protein to its functional mature form and (2) posttranscriptional inhibition of target BDNF gene expression by miRNAs [76]. Hence, the role of miRNAs in BDNF regulation should be considered when developing BDNF-based therapeutic acupuncture treatments for AD.

### 2.4. Depression

#### 2.4.1. BDNF Plays Important Roles in Depression

Depression affects a growing number of patients both physically and mentally. Depression can result in cognitive impairment in addition to mood changes. Severe depression not only results in impaired learning and memory but also compromises the structural and functional integrity of the brain and exhibits maladaptive synaptic plasticity and degenerative changes in the hippocampus and amygdala [104]. Many studies have shown that BDNF is closely related to depression and that BDNF mediates neurogenesis and synaptic plasticity [105]. In animal models of stress, BDNF levels are reduced in both the cortex and the hippocampus [106, 107]. Similarly, the expression of BDNF was significantly decreased in postmortem brain samples of depressed patients [108, 109], whereas the expression of BDNF in the hippocampus of subjects who took antidepressants was higher than that of subjects who did not take antidepressants. Further study revealed that antidepressant-dependent BDNF levels may prevent or minimize hippocampal changes in human samples [110]. Hence, the BDNF imbalance expression in the brain may help to clarify the relationship between neuroplasticity and the pathophysiology of depression.

#### 2.4.2. miRNAs Play Important Roles in Depression

By analysing the above literature, we found that miR-132 and miR-124 participate in neural plasticity. miR-132 dysregulation in major depressive disorder is associated with multiple facets of brain function and structure in the frontolimbic network (the key network for emotional regulation and memory) [111]. Additionally, miR-124 contributes to chronic ultramild stress- (CUMS-) induced dendritic hypotrophy and reduced spine density of dentate gyrus granule neurons, which controls resilience/susceptibility to chronic stress-induced depression-like behaviours [112]. Further research has revealed that miR-124-3p-mediated stress is also related to synaptic plasticity [113]. Similarly, the combined effect of miR-92a and miR-485 on transcription factors, along with histone-modifying enzymes, may have functional relevance by producing changes in gene regulatory networks that modify the neuroplastic capacity of the adult dorsal hippocampus under stress [114]. In the depression model, miR-137 loss-of-function results in altered synaptic transmission and plasticity and anxiety and depression-like behaviour in mice [115]. Moreover, amelioration of depression-like behaviour also involves modulation of the synapse-associated factor miR-134 within the basolateral amygdala [116]. The literature also suggests that late-life depressive symptoms are associated with downregulation of prefrontal cortex miR-484, which is related to synaptic transmission [117]. In addition, miR-99a may be involved in the regulation of hypothalamic synaptic plasticity and might be a potential therapeutic target for peri/postmenopausal depression [118].

#### 2.4.3. miRNAs May Regulate Depression via Influencing BDNF

In in vivo or in vitro rat experiments, miR-206 has been proven to be an important regulator and participator in depression via its direct target gene BDNF [119]. Additionally, inhibition of miR-124 may be a strategy for treating depression by activating the BDNF-TrkB signalling pathway in the hippocampus [120]. Intriguingly, a previous study demonstrated that miR-16 mediates the action of the antidepressant fluoxetine by acting as a micromanager of hippocampal neurogenesis [121]. The 3′-UTR of BDNF was found to be targeted by miR-16 using miRNA analysis software [122]. Hence, the miR-16/BDNF signalling pathway is involved in depressive disorder and seems to be promising [121]. One study that employed in silico approaches, reporter systems, and analysis of endogenous BDNF showed that miR-1, miR-10b, miR-155, and miR-191 directly repress BDNF expression by binding to their predicted sites in the BDNF 3′-UTR [32]. Simultaneously, evidence revealed that BDNF performs antidepressant functions and can be regulated by miR-155 [123]. Thus, miR-155 may affect the depressant status of patients via BDNF, as summarized in Table 4.

#### 2.4.4. Acupuncture Plays a Therapeutic Role in Depression by Regulating the Expression of BDNF and miRNAs

Acupuncture therapy has been shown not only to be an effective treatment modality for depression but also to improve depression-like behaviours and reverse the impairment of LTP [124]. The neuroprotective effects include upregulating the gene and protein expression of BDNF in the hippocampus [125]. Acupuncture markedly increased BDNF protein levels, which provided further evidence supporting its positive effects [126]. As summarized in Table 4, Luo et al. performed EA at acupoints GV20 and GV29 in animals with depression induced by CUMS. Compared with preacupuncture treatment conditions, depression-like behaviours were ameliorated and induced an increase in BDNF expression in the hippocampus after treatment [127]. Acupuncture may exert neuroprotective effects in several nervous system diseases through the modulation of BDNF. Duan et al. further investigated the antidepressant mechanism of EA at GV20 and GV29. According to their results, EA increased BDNF levels by regulating multiple targets in the cyclic adenosine monophosphate response element-binding protein signalling pathway, thereby promoting nerve regeneration [128]. Similarly, Jiang et al. published a study suggesting that the antidepressant effect of acupuncture might be mediated by regulating the DNA methylation and histone modifications of BDNF [129]. Interestingly, Yang et al. revealed that 2 Hz EA plus 5 mg/kg citalopram produced a remarkably increased expression of BDNF in the hippocampus [130]. In addition, in maternally separated depression rat pups, acupuncture stimulation at HT7 significantly increased the BDNF level of the prefrontal cortex [131].

Interestingly, based on previous studies, miR-16 is closely related to depression, and the 3′-UTR of BDNF was found to be targeted by miR-16 [122]. Zhao et al. evaluated the underlying epigenetic mechanism of EA in depression. The CUMS rat model was used, and EA was administered at acupoints GV20 and GV29. After the administration of EA, depression-like behaviours were improved, and high expression of miR-16 in the hippocampus was inhibited as well [132]. Regrettably, this study did not directly explore the relationship between EA regulation of BDNF and neural plasticity. In summary, acupuncture-promoted plasticity protein BDNF expression seems to play an important role in the development of depression. However, further studies are required to investigate the effects of acupuncture on miRNA expression in depression, as acupuncture could target BDNF and related plasticity mechanisms.

## 3. Discussion

We reviewed various studies that have shown neuroplasticity effects caused by regulation of BDNF and miRNAs in different neurodegenerative diseases. The results of the abovementioned studies suggest that the expression levels of BDNF and various miRNAs, which are thought to play significant roles in various diseases, are changed by acupuncture treatment.

Analysing the literature on stroke suggested that miR-219a and miR-134, which are closely related to synaptic plasticity, were downregulated by EA treatment [26, 42]. Similarly, EA increased miR-181b levels in the penumbra and improved neurobehavioural function [46]. miR-494 was downregulated and miR-206 was upregulated in the penumbra [45]. EA increased cerebral blood flow and alleviated neurological impairment in rats. Moreover, upregulated miR-132 suppressed SOX2 in primary neurons after oxygen-glucose deprivation, which promoted neurite outgrowth [43]. Intriguingly, miR-9 responds locally to BDNF. The expression level of miR-9 in the peri-infarct cortex was increased by EA in stroke rat models [44]. However, upregulation of miR-191a-5p exacerbated neuronal injury and partly reversed the neuroprotective effect of EA treatment after ischaemia/repercussion injury [21]. Other miRNAs are also potentially associated with stroke. For instance, miR-124, miR-210, miR-10b-5p, and miR-155 were shown to directly target BDNF [27, 28, 30, 31]. Numerous studies have reported that the expression levels of BDNF in rat brain tissue surrounding the haematoma, the cerebral cortex, the peri-infract cortex, the subventricular zone, the striatum, the hippocampus, etc. are significantly increased after acupuncture [34–41]. Simultaneously, acupuncture has protective and reparative effects on brain tissue, which can improve the symptoms of cerebral infarct, neurological deficits, and motor function in rats. Its mechanism may be related to the upregulation of BDNF and the promotion of nerve cell growth. These changes and distinct roles of many miRNAs may provide an intriguing connection between the effect of acupuncture on stroke and BDNF.

Remarkably, one of the characteristics of PD is the loss of dopaminergic neurons in the substantia nigra. EA therapy may attenuate this loss by promoting the expression of endogenous BDNF [67]. Similarly, the learning and memory abilities of PD rats were significantly improved compared with those of the model group after EA, accompanied by increased BDNF expression levels [66–73], which may underlie the effectiveness of EA in the treatment of PD. Another study investigated whether acupuncture could regulate the expression of miR-124 in the striatum of transgenic mice with PD. According to the study conclusions, the expression levels of miR-124 and BDNF protein were upregulated after treatment [75]. More significantly, many studies have shown that there are complementary sequences among miR-494-3p, miR-30a-5p, miR-7, and miR-210-3p and BDNF [59, 61–63], suggesting that interfering with the expression of these miRNAs could be a strategy for BDNF regulation in PD pathogenesis. Therefore, miRNAs that can directly target BDNF genes might be associated with the potential acupuncture treatment mechanism in PD.

With regard to AD and its link with miRNAs and BDNF, the expression level of miR-132 was increased via BDNF regulation [93, 94]. Furthermore, miR-322, miR-30a-5p, miR-206, miR-195, miR-10a, and miR-163 were identified to target BDNF [16, 61, 96–98]. Interestingly, EA improved learning and memory in AD rats, promoted neurogenesis in AD, and maintained hippocampal LTP to a certain extent. Almost all studies have suggested that EA can increase BDNF expression levels in the brains of AD model animals [100–103]. Hence, the role of miRNAs in BDNF regulation should be considered when developing BDNF-based acupuncture treatment for AD.

Other miRNAs are also potentially associated with depression. For instance, miR-132, miR-124, miR-124-3p, and miR-137 loss-of-function resulted in altered synaptic transmission and plasticity [113, 115]. Similarly, miR-134, miR-92a, and miR-485 are involved in depression brain neuroplastic capacity [114, 116]. Interestingly, miR-206 and miR-155 were shown to directly regulate BDNF in depression studies [32, 119]. The 3′-UTR of BDNF was also targeted by miR-16, which mediates the action of the antidepressant fluoxetine by acting as a micromanager of hippocampal neurogenesis [122]. Most interestingly, numerous studies have shown that EA can not only inhibit the expression of miR-16 in the hippocampus [132] but also increase the levels of BDNF in the brains of depression model rats [126–130]. Therefore, acupuncture promoted synaptic plasticity via BDNF protein expression and regulated a few miRNAs that were found to target BDNF, which seem to play important roles in the development of depression.

Based on the current analysis of the published literature, we summarize that acupuncture treatment seemingly restores the level of BDNF, which is thought to play significant roles in depression and neurodegenerative diseases such as AD, stroke, and PD. Although it should be critically considered that there are methodological and conclusion differences among the studies, the associations are intriguing and worthy of further analysis and study, especially with respect to neuroplasticity.

Major depressive disorder is a highly prevalent psychiatric disorder that is commonly associated with neurodegenerative diseases. In the actual clinical situation, poststroke depression is one of the most common and well-studied phenomena in poststroke patients. Depression worsens the course of poststroke neurological disorders, with poorer functional recovery [133]. Similarly, psychiatric and mood disturbances are common comorbidities with AD and PD. Depressive symptoms increase the overall burden of illness, mainly due to the negative impact on the quality of life of patients (increased disability and morbidity) [134]. In addition, stroke patients also exhibit an increased risk of depression and dementia [135]. A high comorbidity between stroke, AD, PD, and depression suggests there might be similar mechanisms underlying the course of these diseases, and their shared comorbidity mechanism is worth exploring.

Although the structural and functional changes implicated in the relationship between depression and neurodegeneration seem to be highly complex, excitingly, several studies have shown altered BDNF production and secretion in a variety of neurodegenerative diseases as well as in depression [136]. Overall, BDNF is one of the key molecules modulating and linking brain plasticity, and the neuroplasticity hypothesis postulates that the loss of BDNF plays a major role in the pathophysiology of poststroke depression and depression with AD [137, 138]. Similarly, in a short review, while providing evidence of shared biological substrates between PD and depression, neuroplasticity was underscored by the roles of BDNF [139]. Hence, it is possible that depression and neurodegenerative diseases could be improved by a common neuroplasticity mechanism by regulating BDNF expression.

**Figure 1:**
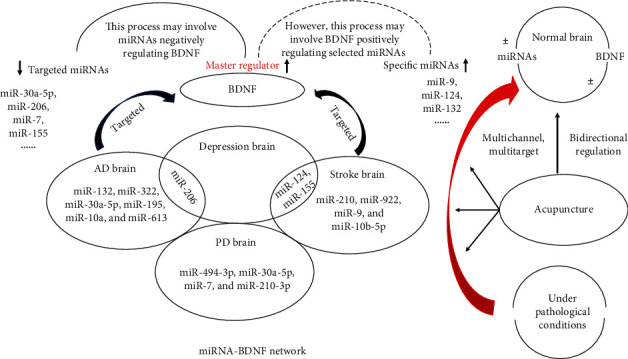
Model illustrating the biological mechanism by which acupuncture regulates the miRNA-BDNF network.

In addition to the coestablished roles of BDNF in modulating neuroplasticity in neurodegenerative diseases and depression, a few other fundamental factors that may have a profound effect in such diseases are currently being explored, such as miRNAs, a class of small noncoding RNAs that can typically bind to the 3′-UTRs of mRNAs to induce repression or degradation. Evidence indicates that the 3′-UTR of BDNF is a significant target of miRNAs, and an in silico analysis suggested that it may have 17 binding sites potentially recognized by as many as 26 miRNAs [61]. In this study, we identified that the expression of BDNF in AD brain neurons is controlled by miR-132, miR-206, miR-30a-5p, miR-195, miR-10a, miR-322, and miR-613; in stroke, the expression of BDNF in brain neurons is controlled by miR-124, miR-210, miR-922, miR-9, miR-10b-5p, and miR-155; in PD brain neurons, the expression of BDNF is controlled by miR-494-3p, miR-30a-5p, miR-7, and miR-210-3p; and in depression brain neurons, it is controlled by miR-206, miR-124, and miR-155. Intriguingly, miR-206 has been proven to be a coregulator in AD and depression. Simultaneously, miR-124 and miR-155 have been shown to be coparticipants in stroke and depression. It is therefore intriguing to speculate that miRNAs might participate in a molecular network involving multiple diseases as the miRNAs that are abundantly expressed seem to overlap between neurodegenerative diseases and depression brain states. We further speculate that BDNF is referred as a “master regulator” because BDNF can be regulated by various miRNAs; thus, the gene expression networks can exert a substantial effect on BDNF. Based on the above multiple regulatory mechanisms, miRNAs build a complex point-to-surface regulatory network, which can not only relate to numerous neurodegenerative diseases and depression states by regulating individual miRNAs but also finely regulate the expression of BDNF by combining several miRNAs. The characteristics of miRNA-BDNF network regulation are highly consistent with the characteristics of multichannel, multitarget, multilevel regulation of acupuncture (Figure 1).

**Figure 2:**
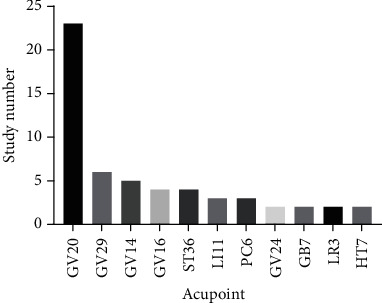
Individual acupoint frequency. If the acupoint is present in no more than two studies, the data is not shown.

**Figure 3:**
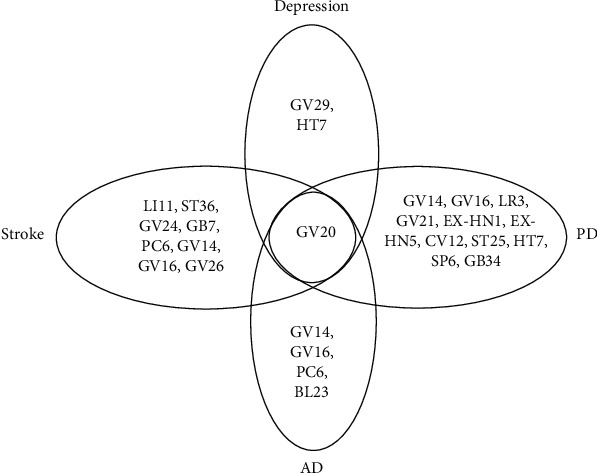
Venn diagram showing acupoints that were differentially administered across the stroke, Alzheimer's disease (AD), Parkinson's disease (PD), and depression relevant studies.

Acupuncture has been practiced in China for over 2000 years to regain the dynamic balance of the organism based on the “meridian theory” as described in the Yellow Emperor's Classic of Internal Medicine. Two types of acupuncture treatment, MA and EA, are distinguished by its treatment method. By manual manipulation or stimulation using a low current and frequency, acupuncture has been shown to modulate neurogenesis and synaptogenesis [140]. According to studies investigating the diseases, the most frequently applied acupoints are GV20, GV29, and GV14 (Figure 2), which are localized on the Governor Vessel (GV). GV runs along the middle of the back and connects with the brain; thus, GV acupoints have always been used for brain and nervous system disorders. Although GV acupoints are frequently used, there are few studies comparing the overlapped molecular outcomes of them with each other or other acupoints among different diseases. In our study, we identified that GV20 elicited the best effects on plasticity (Figure 3), which may contribute to understanding the mechanisms of acupuncture. Overall, the associations are intriguing and worthy of being analysed and studied further.

**Figure 4:**
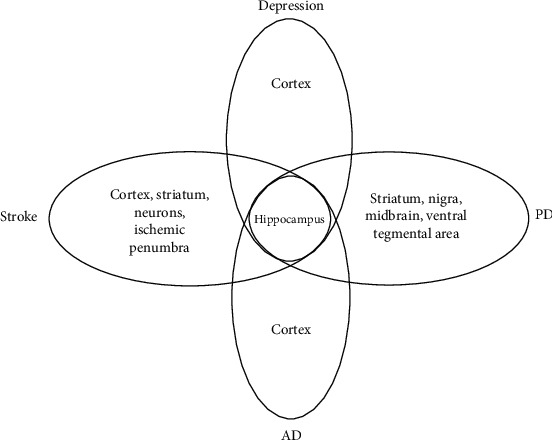
Venn diagram showing brain areas that were differentially selected across the stroke, Alzheimer's disease (AD), Parkinson's disease (PD), and depression relevant studies.

In summary, we propose that it should be critically considered that there are methodological and hypothetical differences between the studies: (1) the regulatory impact of miRNAs on BDNF expression in the brain needs to be strongly considered in the development of therapeutic treatments for neurodegenerative diseases and depression. (2) Although we briefly reviewed the evidence for a positive action of BDNF on miRNA expression and a negative action of miRNAs on BDNF, the miRNA-BDNF pathway may not be a closed loop system, and many other regulatory elements are at play that control specificity of miRNA expression. Hence, this manuscript highlighted the effect of acupuncture and in what way miRNAs have taken part in elucidating mechanism of acupuncture and neuroplasticity. (3) Based on the analysis of the published literature, we summarized that acupuncture treatment seemingly has a bidirectional regulatory ability to restore levels of diverse miRNAs and BDNF to their normal states (Figure 1). (4) Intriguingly, when the same acupoint was used in all four diseases, the underlying effects on the hippocampus may show similar and overlapping molecular outcome among different diseases (Figures 3 and 4). New findings could lead to the discovery of the biological mechanism by which acupuncture regulates the miRNA-BDNF network and could identify the underlying neurodegenerative disease-depression comorbidity mechanism of acupuncture treatment in the near future.

## Figures and Tables

**Table 1:** Deregulated miRNAs and target genes of stroke and summary of related acupuncture literatures.

Study	Species/tissue	miRNA	Result/target genes	
Wang et al. [27]	Stroke patient	miR-124	BDNF	
Zeng et al. [28]	Striatum	miR-210	BDNF	
Liu et al. [29]	Stroke patient	miR-922	The SNP rs7124442 in BDNF 3′-UTR, through affecting the regulatory role of miR-922 in BDNF expression	
Lu et al. [31]	Hippocampus	miR-10b-5p	BDNF	
Varendi et al. [32]	Cellular model	miR-155	BDNF	
Summary of related acupuncture literatures				
Study	Species/tissue	Method/acupoint	Stimulation parameter	Result
Tao et al. [34]	Cortex and striatum	EA at LI11, ST36	1 Hz/20 Hz, 30 min	BDNF ↑
Kim et al. [35]	Striatum and hippocampus	EA at GV20, GV14	2 Hz, 1 mA, 20 min	BDNF ↑
Jiang [36]	Whole brain	EA at GV20, GV16	2 Hz/30 Hz, 30 min, 2 V	BDNF ↑
Kim et al. [37]	Whole brain	EA at GV20, GB7	3 Hz, 5 min	BDNF ↑
Zhou [38]	Cortex	EA at PC6, ST36	2 Hz/30 Hz, 30 min, 5 mA	BDNF ↑
Teng. [39]	Whole brain	MA at GV20-GB7 scalp cave	Needles were turned at a rate of three revolutions per second, twirled 3 times for 5 min, and retained for 30 min	BDNF ↑
Zhang et al. [40]	Hippocampus	MA at nape cluster acupoints	15 min/day	BDNF ↑
Ye et al. [41]	Cortex	EA at LI11, ST36	1 Hz/20 Hz, 30 min, 6 V	BDNF ↑
Liu [42]	Hippocampus	EA at GV20, GV24	2 Hz/20 Hz, 30 min, 6 V	miR-219a ↓
Zhou et al. [21]	Neurons and cortexes	EA at GV20	1 mA, 30 min	miR-191a-5p ↓
Zhao et al. [43]	Ischaemic penumbra	EA at GV20	2 Hz/10 Hz, 1-2 mA, 30 min	miR-132 ↑
Liu et al. [26]	Peri-infarct cortex	EA at GV20, GV24	1 Hz/20 Hz, 0.2 mA, 6 V, 30 min	miR-134 ↓
Liu et al. [44]	Peri-infarct cortex	EA at LI11, ST36	1 Hz/20 Hz, 4 V, 30 min	miR-9 ↑
Zheng et al. [45]	Cortex	EA at GV26, PC6	2 Hz, 3 mA, 1 min	miR-494 ↓, miR-206 ↑
Deng et al. [46]	Ischaemic penumbra	EA at GV20	2 Hz/10 Hz, 1-2 mA, 30 min	miR-181b ↑

MA: manual acupuncture; EA: electroacupuncture.

**Table 2:** Deregulated miRNAs and target genes of Parkinson and summary of related acupuncture literatures.

Study	Species/tissue	miRNA	Result/target genes	
Deng et al. [59]	Cellular model	miR-494-3p	BDNF	
Mellios et al. [61]	Prefrontal cortex	miR-30a-5p	BDNF	
Li et al. [62]	Whole brain	miR-7	BDNF	
Zhang et al. [63]	Cellular model	miR-210-3p	BDNF	
Summary of related acupuncture literatures				
Study	Species/tissue	Method/acupoint	Stimulation parameter	Result
Huang et al. [66]	Nigra	EA at LR3, GV16	100 Hz, 0.5 mA, 30 min	BDNF ↑
Yang et al. [67]	Nigra	EA at GV20, GV16	100 Hz, 1 mA, 30 min	BDNF ↑
Pak et al. [68]	Nigra and striatum	EA at GV20, GV14	2 Hz, 1 mA, 20 min	BDNF ↑
Feng et al. [69]	Whole brain	MA at chorea-tremble controlled zone	20 min/day	BDNF ↑
Zhang [70]	Nigra	EA at GV20, GV21	2 Hz/100 Hz, 1 V, 20 min	BDNF mRNA ↑
Wang [71]	Nigra and striatum	EA at EX-HN1, EX-HN5, CV12, ST25, LR3, HT7, SP6	20 Hz, 1 mA, 20 min	BDNF ↑
Sun et al. [72]	Midbrain and hippocampus	EA at GV20, GV14	0 Hz/100 Hz, 1 mA, 10 min→2 mA, 10 min→3 mA, 10 min	BDNF ↑
Liang et al. [73]	Nigra and ventral tegmental area	EA at GV20, GV14	2 Hz/100 Hz, 30 min 1 mA, 10 min→2 mA, 10 min→3 mA, 10 min	BDNF mRNA ↑, BDNF ↑
Liu [75]	Striatum	MA at GB34, LR3	2 Hz, 1 mA, 20 min	miR-124 ↑

MA: manual acupuncture; EA: electroacupuncture.

**Table 3:** Deregulated miRNAs and target genes of Alzheimer's disease and summary of related electroacupuncture literatures.

Study	Species/tissue	miRNA	Result/target genes	
Vo et al. [93]	Cortical neurons	miR-132	BDNF triggered the rapid induction and persistent expression of mature miR-132	
Numakawa et al. [94]	Cortical neurons	miR-132	BDNF increased levels of synaptic proteins via upregulation of miR-132	
Tian et al. [96]	Hippocampus	miR-206	BDNF	
Mellios et al. [61]	Prefrontal cortex	miR-30a-5p miR-195	Both miR-30a-5p and miR-195 targeted BDNF	
Li et al. [97]	AD patients and mouse model	miR-613	BDNF	
Wu et al. [98]	Neuronal cells	miR-10a	BDNF	
Zhang et al. [16]	Mouse brain	miR-322	BDNF	
Summary of related electroacupuncture literatures				
Study	Species/tissue	Method/acupoint	Stimulation parameter	Result
Wang et al. [100]	Hippocampus	EA at BL23, GV14, PC6	2 Hz, 1 mA, 20 min	BDNF ↑
Zhang et al. [101]	Hippocampus and cortex	EA at GV20, GV16	150 Hz, 15 min	BDNF ↑
Li et al. [102]	Hippocampus and cortex	EA at GV20	2 Hz/15 Hz, 1 mA, 30 min	BDNF ↑
Lin et al. [103]	Hippocampus	EA at GV20	1 Hz/20 Hz, 30 min	BDNF ↑

EA: electroacupuncture.

**Table 4:** Deregulated miRNAs and target genes of depression and summary of related acupuncture literatures.

Study	Species/tissue	miRNA	Result/target genes	
Yang et al. [119]	Hippocampus	miR-206	BDNF	
Wang et al. [120]	Hippocampus	miR-124	miR-124 produced antidepressant-like effects by activating the BDNF-TrkB signalling pathway	
Sun et al. [122]	Cellular model	miR-16	BDNF	
Varendi et al. [32]	Cellular model	miR-155	BDNF	
Summary of related acupuncture literatures				
Study	Species/tissue	Method/acupoint	Stimulation parameter	Result
Zhang et al. [126]	Hippocampus	MA at GV20, GV29	Needles were twirled for 1 min and retained for 10 min	BDNF ↑
Luo et al. [127]	Hippocampus	EA at GV20, GV29	2 Hz, 2 mA, 20 min	BDNF mRNA ↑, BDNF ↑
Duan et al. [128]	Hippocampus	EA at GV20, GV29	2 Hz, 0.6 mA, 30 min	BDNF ↑
Jiang et al. [129]	Hippocampus	MA at GV20, GV29	20 min/day	BDNF ↑
Yang et al. [130]	Hippocampus	EA at GV20 and GV29 combined with citalopram	2 Hz/100 Hz	BDNF ↑
Park et al. [131]	Prefrontal cortex	MA at HT7	Needles were turned at two revolutions per second for 15 s and removed immediately afterward	BDNF ↑
Zhao et al. [132]	Hippocampus	EA at GV20 and GV29	2 Hz, 1 mA, 20 min	miR-16 ↓

MA: manual acupuncture; EA: electroacupuncture.

## References

[1] Mazon J. N., de Mello A. H., Ferreira G. K., Rezin G. T. (2017). The impact of obesity on neurodegenerative diseases. *Life Sciences*.

[2] Erkkinen M. G., Kim M. O., Geschwind M. D. (2018). Clinical neurology and epidemiology of the major neurodegenerative diseases. *Cold Spring Harbor Perspectives in Biology*.

[3] Villas Boas G. R., Boerngen de Lacerda R., Paes M. M. (2019). Molecular aspects of depression: a review from neurobiology to treatment. *European Journal of Pharmacology*.

[4] Réus G. Z., Titus S. E., Abelaira H. M. (2016). Neurochemical correlation between major depressive disorder and neurodegenerative diseases. *Life Sciences*.

[5] Cramer S. C., Sur M., Dobkin B. H. (2011). Harnessing neuroplasticity for clinical applications. *Brain*.

[6] La Rosa C., Parolisi R., Bonfanti L. (2020). Brain structural plasticity: from adult neurogenesis to immature neurons. *Frontiers in Neuroscience*.

[7] Lu B., Nagappan G., Lu Y. (2014). BDNF and synaptic plasticity, cognitive function, and dysfunction. *Handbook of Experimental Pharmacology*.

[8] Leal G., Bramham C. R., Duarte C. B. (2017). BDNF and hippocampal synaptic plasticity. *Vitamins and Hormones*.

[9] Wang Y., Liu H., Zhang B. S., Soares J. C., Zhang X. Y. (2016). Low BDNF is associated with cognitive impairments in patients with Parkinson’s disease. *Parkinsonism & Related Disorders*.

[10] Mang C. S., Campbell K. L., Ross C. J., Boyd L. A. (2013). Promoting neuroplasticity for motor rehabilitation after stroke: considering the effects of aerobic exercise and genetic variation on brain-derived neurotrophic factor. *Physical Therapy*.

[11] Manji H. K., Quiroz J. A., Sporn J. (2003). Enhancing neuronal plasticity and cellular resilience to develop novel, improved therapeutics for difficult-to-treat depression. *Biological Psychiatry*.

[12] Caviedes A., Lafourcade C., Soto C., Wyneken U. (2017). BDNF/NF-kappa B signaling in the neurobiology of depression. *Current Pharmaceutical Design*.

[13] Jin P., Zarnescu D. C., Ceman S. (2004). Biochemical and genetic interaction between the fragile X mental retardation protein and the microRNA pathway. *Nature Neuroscience*.

[14] Saavedra K., Molina-Marquez A. M., Saavedra N., Zambrano T., Salazar L. A. (2016). Epigenetic modifications of major depressive disorder. *International Journal of Molecular Sciences*.

[15] Tavakolizadeh J., Roshanaei K., Salmaninejad A. (2018). MicroRNAs and exosomes in depression: potential diagnostic biomarkers. *Journal of Cellular Biochemistry*.

[16] Zhang J., Liu Z., Pei Y., Yang W., Xie C., Long S. (2018). RETRACTED ARTICLE: MicroRNA-322 cluster promotes tau phosphorylation via targeting brain-derived neurotrophic factor. *Neurochemical Research*.

[17] Ploughman M., Granter-Button S., Chernenko G., Tucker B. A., Mearow K. M., Corbett D. (2005). Endurance exercise regimens induce differential effects on brain-derived neurotrophic factor, synapsin-I and insulin-like growth factor I after focal ischemia. *Neuroscience*.

[18] Chan A., Yan J., Csurhes P., Greer J., McCombe P. (2015). Circulating brain derived neurotrophic factor (BDNF) and frequency of BDNF positive T cells in peripheral blood in human ischemic stroke: effect on outcome. *Journal of Neuroimmunology*.

[19] Ploughman M., Windle V., MacLellan C. L., White N., Dore J. J., Corbett D. (2009). Brain-derived neurotrophic factor contributes to recovery of skilled reaching after focal ischemia in rats. *Stroke*.

[20] Liu X., Feng Z., Du L. (2020). The potential role of micro RNA-124 in cerebral ischemia injury. *International Journal of Molecular Sciences*.

[21] Zhou H., Yang C., Bai F. (2017). Electroacupuncture alleviates brain damage through targeting of neuronal calcium sensor 1 by miR-191a-5p after ischemic stroke. *Rejuvenation Research*.

[22] Chang L., Zhang W., Shi S. (2020). microRNA-195 attenuates neuronal apoptosis in rats with ischemic stroke through inhibiting KLF5-mediated activation of the JNK signaling pathway. *Molecular Medicine*.

[23] Xin H., Li Y., Liu Z. (2013). MiR-133b promotes neural plasticity and functional recovery after treatment of stroke with multipotent mesenchymal stromal cells in rats via transfer of exosome-enriched extracellular particles. *Stem Cells*.

[24] Zhao H., Li G., Zhang S. (2019). Inhibition of histone deacetylase 3 by MiR-494 alleviates neuronal loss and improves neurological recovery in experimental stroke. *Journal of Cerebral Blood Flow and Metabolism*.

[25] Saba R., Storchel P. H., Aksoy-Aksel A. (2012). Dopamine-regulated microRNA MiR-181a controls GluA2 surface expression in hippocampal neurons. *Molecular and Cellular Biology*.

[26] Liu W., Wu J., Huang J. (2017). Electroacupuncture regulates hippocampal synaptic plasticity via miR-134-Mediated LIMK1 function in rats with ischemic stroke. *Neural Plasticity*.

[27] Wang J., Huang Q., Ding J., Wang X. (2019). Elevated serum levels of brain-derived neurotrophic factor and miR-124 in acute ischemic stroke patients and the molecular mechanism. *3 Biotech*.

[28] Zeng L. L., He X. S., Liu J. R., Zheng C. B., Wang Y. T., Yang G. Y. (2016). Lentivirus-mediated overexpression of microRNA-210 improves long-term outcomes after focal cerebral ischemia in mice. *CNS Neuroscience & Therapeutics*.

[29] Liu B., He W., Liu D. (2019). Functional BDNF rs7124442 variant regulated by miR-922 is associated with better short-term recovery of ischemic stroke. *Therapeutics and Clinical Risk Management*.

[30] Wang L., Liu W., Zhang Y. (2020). Dexmedetomidine had neuroprotective effects on hippocampal neuronal cells via targeting lncRNA SHNG16 mediated microRNA-10b-5p/BDNF axis. *Molecular and Cellular Biochemistry*.

[31] Lu Y., Huang Z., Hua Y., Xiao G. (2018). Minocycline promotes BDNF expression of N2a cells via inhibition of miR-155-mediated repression after oxygen-glucose deprivation and reoxygenation. *Cellular and Molecular Neurobiology*.

[32] Varendi K., Kumar A., Harma M. A., Andressoo J. O. (2014). miR-1, miR-10b, miR-155, and miR-191 are novel regulators of BDNF. *Cellular and Molecular Life Sciences*.

[33] Dajas-Bailador F., Bonev B., Garcez P., Stanley P., Guillemot F., Papalopulu N. (2012). MicroRNA-9 regulates axon extension and branching by targeting Map1b in mouse cortical neurons. *Nature Neuroscience*.

[34] Tao J., Zheng Y., Liu W. (2016). Electro-acupuncture at LI11 and ST36 acupoints exerts neuroprotective effects via reactive astrocyte proliferation after ischemia and reperfusion injury in rats. *Brain Research Bulletin*.

[35] Kim Y. R., Ahn S. M., Pak M. E. (2018). Potential benefits of mesenchymal stem cells and electroacupuncture on the trophic factors associated with neurogenesis in mice with ischemic stroke. *Scientific Reports*.

[36] Jiang C. (2006). *From BDNF, NGF and Nestin angle to study the protection and repair mechanism of electroacupuncture on the neural damage of brain ischemia*.

[37] Kim M. W., Chung Y. C., Jung H. C. (2012). Electroacupuncture enhances motor recovery performance with brain-derived neurotrophic factor expression in rats with cerebral infarction. *Acupuncture in Medicine*.

[38] Zhou L. (2019). *Effects of electroacupuncture on the expression of BDNF, Sema3A, and NRP-1 in rats with focal cerebral infarction*.

[39] Teng W. (2009). *The experimental influences of Baihui-Qubin acupuncture on brain-derived neurotrophic factor expression of rats with intracerebral hemorrhage*.

[40] Xiao W., Zhang X., Wang Z., Wang Y., Guo X., Ling H. E. (2014). Effect of nape cluster acupuncture on BDNF, NGF and neurobehaviors in rats with post-ischemic stroke sequelae. *Shanghai Journal of Acupuncture and Moxibustion*.

[41] Ye X., Jiang Y., You Y. (2014). The effect of electroacupuncture the expression of brain-derived neurotrophic factor in cerebral ischemia-reperfusion injury model rats. *Chinese Journal of Rehabilitation Medicine*.

[42] Liu W. (2017). *Based on miRNAs mediating synaptic plasticity to investigate the mechanism of electroacupuncture improving learning and memory in a rat model of ischemic stroke*.

[43] Zhao X., Bai F., Zhang E. (2018). Electroacupuncture improves neurobehavioral function through targeting of SOX2-mediated axonal regeneration by microRNA-132 after ischemic stroke. *Frontiers in Molecular Neuroscience*.

[44] Liu W., Wang X., Zheng Y. (2016). Electroacupuncture inhibits inflammatory injury by targeting the miR-9-mediated NF-*κ*B signaling pathway following ischemic stroke. *Molecular Medicine Reports*.

[45] Zheng H. Z., Jiang W., Zhao X. F. (2016). Electroacupuncture induces acute changes in cerebral cortical miRNA profile, improves cerebral blood flow and alleviates neurological deficits in a rat model of stroke. *Neural Regeneration Research*.

[46] Deng B., Bai F., Zhou H. (2016). Electroacupuncture enhances rehabilitation through miR-181b targeting PirB after ischemic stroke. *Scientific Reports*.

[47] Costa A., Peppe A., Carlesimo G. A. (2015). Brain-derived neurotrophic factor serum levels correlate with cognitive performance in Parkinson’s disease patients with mild cognitive impairment. *Frontiers in Behavioral Neuroscience*.

[48] Howells D. W., Porritt M. J., Wong J. Y. F. (2000). Reduced BDNF mRNA expression in the Parkinson’s disease substantia nigra. *Experimental Neurology*.

[49] Scalzo P., Kümmer A., Bretas T. L., Cardoso F., Teixeira A. L. (2010). Serum levels of brain-derived neurotrophic factor correlate with motor impairment in Parkinson’s disease. *Journal of Neurology*.

[50] Gyárfás T., Knuuttila J., Lindholm P., Rantamäki T., Castrén E. (2010). Regulation of brain-derived neurotrophic factor (BDNF) and cerebral dopamine neurotrophic factor (CDNF) by anti-parkinsonian drug therapy in vivo. *Cellular and Molecular Neurobiology*.

[51] Hirsch M. A., Iyer S. S., Sanjak M. (2016). Exercise-induced neuroplasticity in human Parkinson’s disease: what is the evidence telling us?. *Parkinsonism & Related Disorders*.

[52] Gillardon F., Mack M., Rist W. (2008). MicroRNA and proteome expression profiling in early-symptomatic *α*-synuclein(A30P)-transgenic mice. *Proteomics. Clinical Applications*.

[53] Goh S. Y., Chao Y. X., Dheen S. T., Tan E. K., Tay S. S. (2019). Role of microRNAs in Parkinson’s disease. *International Journal of Molecular Sciences*.

[54] Lyu G., Guan Y., Zhang C. (2018). TGF-*β* signaling alters H4K20me3 status via miR-29 and contributes to cellular senescence and cardiac aging. *Nature Communications*.

[55] Gu Q. H., Yu D., Hu Z. (2015). miR-26a and miR-384-5p are required for LTP maintenance and spine enlargement. *Nature Communications*.

[56] Cohen J. E., Lee P. R., Chen S., Li W., Fields R. D. (2011). MicroRNA regulation of homeostatic synaptic plasticity. *Proceedings of the National Academy of Sciences*.

[57] Qian Y., Song J., Ouyang Y. (2017). Advances in roles of miR-132 in the nervous system. *Frontiers in Pharmacology*.

[58] Geng L., Zhang T., Liu W., Chen Y. (2018). miR-494-3p modulates the progression of in vitro and in vivo Parkinson’s disease models by targeting SIRT3. *Neuroscience Letters*.

[59] Deng C., Zhu J., Yuan J., Xiang Y., Dai L. (2020). Pramipexole inhibits MPP+-induced neurotoxicity by miR-494-3p/BDNF. *Neurochemical Research*.

[60] Schwienbacher C., Foco L., Picard A. (2017). Plasma and white blood cells show different miRNA expression profiles in Parkinson’s disease. *Journal of Molecular Neuroscience*.

[61] Mellios N., Huang H. S., Grigorenko A., Rogaev E., Akbarian S. (2008). A set of differentially expressed miRNAs, including miR-30a-5p, act as post-transcriptional inhibitors of BDNF in prefrontal cortex. *Human Molecular Genetics*.

[62] Li B., Jiang Y., Xu Y., Li Y., Li B. (2019). Identification of miRNA-7 as a regulator of brain-derived neurotrophic factor/*α*-synuclein axis in atrazine-induced Parkinson’s disease by peripheral blood and brain microRNA profiling. *Chemosphere*.

[63] Zhang S., Chen S., Liu A. (2018). Inhibition of BDNF production by MPP + through up-regulation of miR-210-3p contributes to dopaminergic neuron damage in MPTP model. *Neuroscience Letters*.

[64] Wang H., Liang X., Wang X., Luo D., Jia J., Wang X. (2013). Electro-acupuncture stimulation improves spontaneous locomotor hyperactivity in MPTP intoxicated mice. *PLoS One*.

[65] Wang S., Fang J., Ma J. (2013). Electroacupuncture-regulated neurotrophic factor mRNA expression in the substantia nigra of Parkinson’s disease rats. *Neural Regeneration Research*.

[66] Huang P., Ma J., Wang Y., Gan S., Li H., Lei T. (2010). Effect of Taichong and Fengfu on learning and memory ability and brain-derived neurotrophic factor in a rat model of Parkinson’s disease. *Traditional Chinese Medicine Journal*.

[67] Yang D., Cai M., Chen X., Ou H. (2014). Effect of electroacupuncture scalp points on the expression of the brain-derived neurotrophic factor in substantia nigra in the Parkinson’s disease rat model. *Journal of Zhejiang Chinese Medical University*.

[68] Pak M. E., Ahn S. M., Jung D. H. (2020). Electroacupuncture therapy ameliorates motor dysfunction via brain-derived neurotrophic factor and glial cell line-derived neurotrophic factor in a mouse model of Parkinson’s disease. *The Journals of Gerontology. Series A, Biological Sciences and Medical Sciences*.

[69] Feng J., Sun H. M., Wang Y. Y. (2014). Influences of needling chorea-tremble controlled zone on expressions of dopaminergic neurons and BDNF in mice with Parkinson’s disease. *Journal of Beijing University of Traditional Chinese Medicine*.

[70] Zhang J. (2010). *Experimental studies on the intervention of electroacupuncture ‘Baihui through Qianding’ in Parkinson’s disease mice*.

[71] Wang H. (2018). *Effects of different electricity on serum and black striatum BDNF in Parkinsonism model rats*.

[72] Sun M., Wang K., Yu Y. (2016). Electroacupuncture alleviates depressive-like symptoms and modulates BDNF signaling in 6-hydroxydopamine rats. *Evidence-based Complementary and Alternative Medicine*.

[73] Liang X.-B., Liu X.-Y., Li F.-Q. (2002). Long-term high-frequency electro-acupuncture stimulation prevents neuronal degeneration and up-regulates BDNF mRNA in the substantia nigra and ventral tegmental area following medial forebrain bundle axotomy. *Molecular Brain Research*.

[74] Sun Y., Luo Z.-M., Guo X.-M., Su D.-F., Liu X. (2015). An updated role of microRNA-124 in central nervous system disorders: a review. *Frontiers in Cellular Neuroscience*.

[75] Liu Y. (2018). *Effect of acupuncture on behaviour and expression of miRNA-124 in the striatum of transgenic mice with Parkinson’s disease*.

[76] Keifer J., Zheng Z., Ambigapathy G. (2015). A microRNA-BDNF negative feedback signaling loop in brain: implications for Alzheimer’s disease. *Microrna*.

[77] Arora S., Sharma D., Singh J. (2020). GLUT-1: an effective target to deliver brain-derived neurotrophic factor gene across the blood brain barrier. *ACS Chemical Neuroscience*.

[78] Lau P., Bossers K., Janky R.’s. (2013). Alteration of the microRNAnetwork during the progression of Alzheimer’s disease. *EMBO Molecular Medicine*.

[79] Baby N., Alagappan N., Dheen S. T., Sajikumar S. (2020). MicroRNA-134-5p inhibition rescues long-term plasticity and synaptic tagging/capture in an A*β* (1-42)-induced model of Alzheimer’s disease. *Aging Cell*.

[80] Remenyi J., van den Bosch M. W. M., Palygin O. (2013). miR-132/212 knockout mice reveal roles for these miRNAs in regulating cortical synaptic transmission and plasticity. *PLoS One*.

[81] Xu N., Li A.-D., Ji L.-L., Ye Y., Wang Z.-Y., Tong L. (2019). miR-132 regulates the expression of synaptic proteins in APP/PS1 transgenic mice through C1q. *European Journal of Histochemistry*.

[82] Song J., Kim Y. K. (2017). Identification of the role of miR-142-5p in Alzheimer’s disease by comparative bioinformatics and cellular analysis. *Frontiers in Molecular Neuroscience*.

[83] Wang X., Liu D., Huang H. Z. (2018). A novel microRNA-124/PTPN1 signal pathway mediates synaptic and memory deficits in Alzheimer’s disease. *Biological Psychiatry*.

[84] Rodriguez‐Ortiz C. J., Prieto G. A., Martini A. C. (2020). miR-181a negatively modulates synaptic plasticity in hippocampal cultures and its inhibition rescues memory deficits in a mouse model of Alzheimer’s disease. *Aging Cell*.

[85] Qian Q., Zhang J., He F.-P. (2019). Down-regulated expression of microRNA-338-5p contributes to neuropathology in Alzheimer’s disease. *The FASEB Journal*.

[86] Rodriguez-Ortiz C. J., Baglietto-Vargas D., Martinez-Coria H., LaFerla F. M., Kitazawa M. (2014). Upregulation of miR-181 decreases c-Fos and SIRT-1 in the hippocampus of 3xTg-AD mice. *Journal of Alzheimer's Disease*.

[87] Yan M.-L., Zhang S., Zhao H.-M. (2020). MicroRNA-153 impairs presynaptic plasticity by blocking vesicle release following chronic brain hypoperfusion. *Cell Communication and Signaling*.

[88] Xu Y., Chen P., Wang X., Yao J., Zhuang S. (2018). miR-34a deficiency in APP/PS1 mice promotes cognitive function by increasing synaptic plasticity via AMPA and NMDA receptors. *Neuroscience Letters*.

[89] Kao Y. C., Wang I. F., Tsai K. J. (2018). miRNA-34c overexpression causes dendritic loss and memory decline. *International Journal of Molecular Sciences*.

[90] Sarkar S., Jun S., Rellick S., Quintana D. D., Cavendish J. Z., Simpkins J. W. (2016). Expression of microRNA-34a in Alzheimer’s disease brain targets genes linked to synaptic plasticity, energy metabolism, and resting state network activity. *Brain Research*.

[91] Lee S. T., Chu K., Jung K. H. (2012). miR-206 regulates brain-derived neurotrophic factor in Alzheimer disease model. *Annals of Neurology*.

[92] Cogswell J. P., Ward J., Taylor I. A. (2008). Identification of miRNA changes in Alzheimer’s disease brain and CSF yields putative biomarkers and insights into disease pathways. *Journal of Alzheimer's Disease*.

[93] Vo N., Klein M. E., Varlamova O. (2005). From the cover: a cAMP-response element binding protein-induced microRNA regulates neuronal morphogenesis. *Proceedings of the National Academy of Sciences*.

[94] Numakawa T., Richards M., Adachi N., Kishi S., Kunugi H., Hashido K. (2011). MicroRNA function and neurotrophin BDNF. *Neurochemistry International*.

[95] Xie B., Liu Z., Jiang L. (2017). Increased serum miR-206 level predicts conversion from amnestic mild cognitive impairment to Alzheimer’s disease: a 5-year follow-up study. *Journal of Alzheimer's Disease*.

[96] Tian N., Cao Z., Zhang Y. (2014). MiR-206 decreases brain-derived neurotrophic factor levels in a transgenic mouse model of Alzheimer’s disease. *Neuroscience Bulletin*.

[97] Li W., Li X., Xin X., Kan P. C., Yan Y. (2016). MicroRNA-613 regulates the expression of brain-derived neurotrophic factor in Alzheimer’s disease. *Bioscience Trends*.

[98] Wu B. W., Wu M. S., Guo J. D. (2018). Effects of microRNA-10a on synapse remodeling in hippocampal neurons and neuronal cell proliferation and apoptosis through the BDNF-TrkB signaling pathway in a rat model of Alzheimer’s disease. *Journal of Cellular Physiology*.

[99] Song L., Shu-guang Y., Ting H. (2006). Effects of electroacupuncture on synaptic plasticity of hippocampal neurons in model rats with Alzheimer disease. *Chinese Journal of Clinical Rehabilitation*.

[100] Wang X., Shen F., Kong L. (2015). Effects of low frequency electroacupuncture on expression of BDNF in hippocampus of Alzheimer’s disease model rats. *Journal of Hubei University of Chinese Medicine*.

[101] Zhang D., Sun Y., Wang L. (2011). The influence of electroacupuncture Baihui and Fengfu on behavior and the expression of brain BDNF in rats model of learning and memory impairement. *Acta Chinese Medicine and Pharmacology*.

[102] Li X., Guo F., Zhang Q. (2014). Electroacupuncture decreases cognitive impairment and promotes neurogenesis in the APP/PS1 transgenic mice. *BMC Complementary and Alternative Medicine*.

[103] Lin R., Chen J., Li X. (2016). Electroacupuncture at the Baihui acupoint alleviates cognitive impairment and exerts neuroprotective effects by modulating the expression and processing of brain-derived neurotrophic factor in APP/PS1 transgenic mice. *Molecular Medicine Reports*.

[104] Mahati K., Bhagya V., Christofer T., Sneha A., Shankaranarayana Rao B. S. (2016). Enriched environment ameliorates depression-induced cognitive deficits and restores abnormal hippocampal synaptic plasticity. *Neurobiology of Learning and Memory*.

[105] Groves J. O. (2007). Is it time to reassess the BDNF hypothesis of depression?. *Molecular Psychiatry*.

[106] Duman R. S., Monteggia L. M. (2006). A neurotrophic model for stress-related mood disorders. *Biological Psychiatry*.

[107] Molteni R., Calabrese F., Cattaneo A. (2009). Acute stress responsiveness of the neurotrophin BDNF in the rat hippocampus is modulated by chronic treatment with the antidepressant duloxetine. *Neuropsychopharmacology*.

[108] Guilloux J. P., Douillard-Guilloux G., Kota R. (2012). Molecular evidence for BDNF- and GABA-related dysfunctions in the amygdala of female subjects with major depression. *Molecular Psychiatry*.

[109] Tripp A., Oh H., Guilloux J. P., Martinowich K., Lewis D. A., Sibille E. (2012). Brain-derived neurotrophic factor signaling and subgenual anterior cingulate cortex dysfunction in major depressive disorder. *The American Journal of Psychiatry*.

[110] Chen B., Dowlatshahi D., MacQueen G. M., Wang J. F., Young L. T. (2001). Increased hippocampal BDNF immunoreactivity in subjects treated with antidepressant medication. *Biological Psychiatry*.

[111] Qi S., Yang X., Zhao L. (2018). MicroRNA132 associated multimodal neuroimaging patterns in unmedicated major depressive disorder. *Brain*.

[112] Higuchi F., Uchida S., Yamagata H. (2016). Hippocampal microRNA-124 enhances chronic stress resilience in mice. *The Journal of Neuroscience*.

[113] Roy B., Dunbar M., Shelton R. C., Dwivedi Y. (2017). Identification of microRNA-124-3p as a putative epigenetic signature of major depressive disorder. *Neuropsychopharmacology*.

[114] Muñoz-Llanos M., García-Pérez M. A., Xu X. (2018). MicroRNA profiling and bioinformatics target analysis in dorsal hippocampus of chronically stressed rats: relevance to depression pathophysiology. *Frontiers in Molecular Neuroscience*.

[115] Yan H. L., Sun X. W., Wang Z. M. (2019). MiR-137 deficiency causes anxiety-like behaviors in mice. *Frontiers in Molecular Neuroscience*.

[116] Yu H., Fan C., Yang L. (2018). Ginsenoside Rg1 prevents chronic stress-induced depression-like behaviors and neuronal structural plasticity in rats. *Cellular Physiology and Biochemistry*.

[117] Wingo T. S., Yang J., Fan W. (2020). Brain microRNAs associated with late-life depressive symptoms are also associated with cognitive trajectory and dementia. *NPJ Genomic Medicine*.

[118] Yang J., Zhang L., Cao L. L. (2019). MicroRNA-99a is a potential target for regulating hypothalamic synaptic plasticity in the peri/postmenopausal depression model. *Cells*.

[119] Yang X., Yang Q., Wang X. (2014). MicroRNA expression profile and functional analysis reveal that miR-206 is a critical novel gene for the expression of BDNF induced by ketamine. *Neuromolecular Medicine*.

[120] Wang S.-S., Mu R.-H., Li C.-F. (2017). MicroRNA-124 targets glucocorticoid receptor and is involved in depression-like behaviors. *Progress in Neuro-Psychopharmacology and Biological Psychiatry*.

[121] Launay J. M., Mouillet-Richard S., Baudry A., Pietri M., Kellermann O. (2011). Raphe-mediated signals control the hippocampal response to SRI antidepressants via miR-16. *Translational Psychiatry*.

[122] Sun Y.-X., Yang J., Wang P.-Y., Li Y.-J., Xie S.-Y., Sun R.-P. (2013). Cisplatin regulates SH-SY5Y cell growth through downregulation of BDNF via miR-16. *Oncology Reports*.

[123] Xu N., Meng H., Liu T. (2017). Blueberry phenolics reduce gastrointestinal infection of patients with cerebral venous thrombosis by improving depressant-induced autoimmune disorder via miR-155-mediated brain-derived neurotrophic factor. *Frontiers in Pharmacology*.

[124] She Y., Xu J., Duan Y. (2015). Possible antidepressant effects and mechanism of electroacupuncture in behaviors and hippocampal synaptic plasticity in a depression rat model. *Brain Research*.

[125] Lin D., Wu Q., Lin X. (2015). Brain-derived neurotrophic factor signaling pathway: modulation by acupuncture in telomerase knockout mice. *Alternative Therapies in Health and Medicine*.

[126] Zhang X., Song Y., Bao T. (2016). Antidepressant-like effects of acupuncture involved the ERK signaling pathway in rats. *BMC Complementary and Alternative Medicine*.

[127] Luo T., Tian H., Song H. (2020). Possible involvement of tissue plasminogen activator/brain-derived neurotrophic factor pathway in anti-depressant effects of electroacupuncture in chronic unpredictable mild stress-induced depression in rats. *Frontiers in Psychiatry*.

[128] Duan D. M., Tu Y., Liu P., Jiao S. (2016). Antidepressant effect of electroacupuncture regulates signal targeting in the brain and increases brain-derived neurotrophic factor levels. *Neural Regeneration Research*.

[129] Jiang H., Zhang X., Lu J. (2018). Antidepressant-like effects of acupuncture-insights from DNA methylation and histone modifications of brain-derived neurotrophic factor. *Frontiers in Psychiatry*.

[130] Yang J., Pei Y., Pan Y. L. (2013). Enhanced antidepressant-like effects of electroacupuncture combined with citalopram in a rat model of depression. *Evidence-based Complementary and Alternative Medicine*.

[131] Park H., Yoo D., Kwon S. (2012). Acupuncture stimulation at HT7 alleviates depression-induced behavioral changes via regulation of the serotonin system in the prefrontal cortex of maternally-separated rat pups. *The Journal of Physiological Sciences*.

[132] Zhao J., Tian H., Song H. (2019). Effect of electroacupuncture on reuptake of serotonin via miRNA-16 expression in a rat model of depression. *Evidence-based Complementary and Alternative Medicine*.

[133] Loubinoux I., Kronenberg G., Endres M. (2012). Post-stroke depression: mechanisms, translation and therapy. *Journal of Cellular and Molecular Medicine*.

[134] Galts C. P. C., Bettio L. E. B., Jewett D. C. (2019). Depression in neurodegenerative diseases: common mechanisms and current treatment options. *Neuroscience and Biobehavioral Reviews*.

[135] Filipska K., Wisniewski A., Biercewicz M., Slusarz R. (2020). Are depression and dementia a common problem for stroke older adults? A review of chosen epidemiological studies. *Psychiatric Quarterly*.

[136] Pluchino N., Russo M., Santoro A. N., Litta P., Cela V., Genazzani A. R. (2013). Steroid hormones and BDNF. *Neuroscience*.

[137] Fukuchi M. (2020). Identifying inducers of BDNF gene expression from pharmacologically validated compounds; antipyretic drug dipyrone increases BDNF mRNA in neurons. *Biochemical and Biophysical Research Communications*.

[138] Chen C., Dong Y., Liu F. (2020). A study of antidepressant effect and mechanism on intranasal delivery of BDNF-HA2TAT/AAV to rats with post-stroke depression. *Neuropsychiatric Disease and Treatment*.

[139] Tizabi Y., Getachew B., Csoka A. B., Manaye K. F., Copeland R. L. (2019). Novel targets for parkinsonism-depression comorbidity. *Progress in Molecular Biology and Translational Science*.

[140] Xiao L. Y., Wang X. R., Yang Y. (2018). Applications of acupuncture therapy in modulating plasticity of central nervous system. *Neuromodulation*.

